# Understanding allosteric interactions in hMLKL protein that modulate necroptosis and its inhibition

**DOI:** 10.1038/s41598-019-53078-5

**Published:** 2019-11-14

**Authors:** Nupur Bansal, Simone Sciabola, Govinda Bhisetti

**Affiliations:** Biotherapeutic and Medicinal Sciences, Biogen, 225 Binney Street, Cambridge, MA 02142 United States of America

**Keywords:** Computational chemistry, Kinases

## Abstract

Mixed Lineage Kinase domain-Like (MLKL), a key player in necroptosis, is a multi-domain protein with an N-terminal 4 helical bundle (4HB) and a pseudokinase domain (PsK) connected by brace helices. Phosphorylation of PsK domain of MLKL is a key step towards oligomerization of 4HB domain that causes cell death. Necrosulfonamide (NSA) binds to the 4HB domain of MLKL to inhibit necroptosis. To understand the molecular details of MLKL function and it’s inhibition, we have performed a molecular dynamic study on hMLKL protein in apo, phosphorylated and NSA-bound states for a total 3 μs simulation time. Our simulations show increased inter-domain flexibility, increased rigidification of the activation loop and increased alpha helical content in the brace helix region revealing a form of monomeric hMLKL necessary for oligomerization upon phosphorylation as compared to apo state. NSA binding disrupts this activated form and causes two main effects on hMLKL conformation: (1) locking of the relative orientation of 4HB and PsK domains by the formation of several new interactions and (2) prevention of key 4HB residues to participate in cross-linking for oligomer formation. This new understanding of the effect of hMLKL conformations on phosphorylation and NSA binding suggest new avenues for designing effective allosteric inhibitors of hMLKL.

## Introduction

Necroptosis is a non-apoptotic, caspase free and kinase dependent programmed cell death which leads to plasma membrane rupture and cell lysis^1–4^. Mixed Lineage Kinase domain-Like (MLKL) protein is the most critical downstream effector of TNF induced necroptotic pathway^5–7^. The core necroptotic pathway relies on the hetero oligomerization of receptor interacting protein kinase (RIPK) 1 and 3 to form necrosome^8^. RIPK3 in turn activates MLKL protein by phosphorylation at Thr357 and Ser358^6,9^. It has been hypothesized that the activated MLKL undergoes conformational changes that lead to its oligomerization and permeation through the cell membrane to cause cell death^10–12^. Although phosphorylation of PsK domain triggers activation in both human and mouse MLKL, there are significant differences in the activation mechanisms between mouse and human MLKL^13^. It has also been established that IP-Kinases play a regulatory role in MLKL activation potentially influencing oligomerization and/or membrane recruitment^14^. While oligomerization of MLKL has been established as a central element in necroptosis, the molecular details of oligomer formation remain elusive^9,10,12,13,15–18^.

Structurally, MLKL comprises of a N-terminal 4-helix bundle (4HB) domain and a C-terminal pseudokinase (PsK) domain linked together by two brace helices^5^ (Fig. S1). The pseudokinase domain is termed so as it lacks the kinase catalytic activity. The DFG loop in MLKL is replaced with GFE and the catalytic loop motif HRD is replaced with HGK making it catalytically dead^5,19,20^. Activation of MLKL is known to cause unleashing of the N-terminal 4HB domain, which leads to its oligomerization and permeation of 4HB through the cell membrane eventually leading to cell death^9–12^. Brace helices have been shown to mediate the phosphorylation signal from PsK to N-terminal 4HB domain^21,22^. Also, it has been proposed that inositol phosphate metabolites (IP_6_) bind to N-terminal and brace regions to regulate MLKL function. But alternative regulatory mechanisms such as membrane recruitment are also proposed for IP_6_^14^. In any case, it is clear that phosphorylation of the PsK domain transmits the signal for oligomerization to 4HB domain; the details of this molecular communication are not well-understood.

Studies have been conducted to design MLKL inhibitors for blocking necroptotic pathway. Ma *et al*. identified several ATP-pocket binders for hMLKL but they observed that these binders have no direct effect on MLKL and therefore on necroptosis^23^. The only known effective inhibitor of necroptosis directly binding to MLKL is necrosulfonamide (NSA)^6^. Exactly how NSA binding to the N-terminal domain blocks necroptosis is also not clearly elucidated. Our goal in this molecular dynamics study is to unravel details of conformational changes that may occur upon NSA binding to the monomeric hMLKL that prevent oligomerization and unleashing of 4HB domain.

We performed classical molecular dynamic (MD) simulations on a full length monomeric hMLKL protein in three different states - apo, phosphorylated (activated) and Necrosulfonamide (NSA) bound (inhibitor bound) for a total of 1 μs for each state to delineate the molecular details of activation of MLKL by phosphorylation and it’s inhibition by NSA. Our simulations data provide novel insights into the dynamical and structural changes observed in hMLKL upon activation and inhibition. We also rationalize some previously unexplained phenomena associated with NSA binding. This understanding of the role of different structural domains of MLKL can help in designing better hMLKL targeting drugs. It is clear from our results that inhibitors that stabilize the locked conformation of MLKL will be effective in blocking oligomerization and necroptosis.

## Methods

### Full length model of human MLKL

Crystal structures of PsK domains of hMLKL^19^, NMR structure of human 4HB domain^24^, but not the full-length hMLKL structure are available. Also, the crystal structure of full-length mouse MLKL structure is available^5^. We utilized available crystal structures to build a full length chimeric model of monomeric hMLKL structure for performing MD simulations. The full length chimeric model of human monomeric MLKL protein was generated by using published crystal structures of human MLKL kinase domain (PDBID:4MWI), NMR structure of 4HB (PDB:2MSV) and full-length mouse crystal structure (PDB:4BTF) as templates. All the input structures were prepared by using Protein preparation wizard utility in Maestro using Schrödinger Suite molecular modeling package^25^. All the missing loops and side chains were filled during protein preparation. The mutation in PDB:2MSV was reverted back to wild type construct. Chimeric model was built using advanced homology model panel in Maestro using Prime^26^. 4HB N-terminal domain was taken from PDB: 2MSV and the PsK domain (residue 193–471) was taken from PDB: 4MWI. The intermediate structure containing the brace helices was taken from mouse PDB: 4BTF. Mouse and human MLKL amino acid sequences were aligned to build a complete model of human MLKL. Steric clashes and other unfavorable interactions observed in the protein reliability report were resolved by running restrained minimization in the protein preparation wizard utility. ATP was added to the model by extracting the ATP coordinates from the structure of ATP bound Kinase in PDB:1ATP^27^ after it was aligned with the MLKL model. The ATP-bound full length MLKL model was prepared for Molecular Dynamics simulations using protein preparation wizard followed by minimization using Macromodel with default settings^28^. MLKL is known to bind ATP in the absence of metal ions^5^, therefore no metal ions were included in the model. Thr357 and Ser358 residues were phosphorylated by using the build panel in Maestro. NSA was added to the phosphorylated model by performing covalent docking mimicking Michaels reaction^29^. The final three models were fully minimized by using Macromodel with default settings^30^.

### MD simulations

The protonation states of the protein residues at pH 7.0 were estimated using the protein Prepare function in HTMD^31^ (version 1.13.7). The structures were solvated with water molecules in a cubic box of side equal to 2 × (**m** + 5) Å, where **m** is the maximum distance of any atom in the structure and its center, using the solvate function of HTMD (version 1.13.7). Finally, the systems were built and neutralized using the amber.build function in HTMD^31,32^ (version 1.13.7). Na^ + ^and Cl^−^ ions were added to neutralize the system and make a salt concentration of 0.1 M. The following parameters were used for building of the systems: ff14SB^33^ for protein, TIP3P^34^ for the water model, Na^ + ^and Cl^−^ ions from Joung and Cheatham^35^, ATP from Meagher *et al*.^36^, and phosphorylated serine (SEP) and threonine (TPO) from Khoury *et al*.^37^.

For the necrosulfonamide conjugated to Cys86, from the initial structure, we have constructed a residue called CNS and created the parameters for it. For the calculation of the charges, the CNS residue capped with acetyl (ACE) and methylamine (NME) was used as the initial structure (Fig. S2 in the Supplementary Information). The initial charges were first calculated for the CNS residue using the Gasteiger model^38^, and then the charges for the ACE, NME and cysteine up to atom S10 (see Fig. S2; atoms H0, C1, H2, H3, C4, O5, N6, H7, C8, C9, S10, H49, H50, H51, C52, O53, N54, H55, C56, H57, H58, H59) were changed to match the charges of ACE-CYX-NME in ff14SB, where CYX is the cysteine residue for a disulfide bridge. After this, the structure passed two blocks of QM calculations: first, a geometry optimization block with up to 250 steps at the B3LYP/6–31 G* level; second, a QM ESP block also at the B3LYP/6–31 G* level, with the ESP evaluations being organized into five layers of grids that are 1.4, 1.6, 1.8, 2.0, and 2.2 times the van der Waals radii, following the procedure of Amber force-field development^39^. During this calculation, the charges of the above-mentioned 22 atoms were kept fixed. The final charges were used for the CNS residue. For the Lennard-Jones and bonded parameters, ff14SB was used for all the above mentioned 22 atoms, while GAFF2 parameters were used for the other atoms. The parameters for bonded terms were also derived from GAFF2, except for the C9-S10-C11 angle terms which were taken from the ff14SB parameters for methionine (CT-S-2C angle).

All the systems were simulated using ACEMD^40^ at 300 K. Each system underwent an equilibration protocol of 3 ns followed by a production protocol of 1μs, as described in HTMD Protocols^31^, using HTMD^32^ version 1.13.8. The default equilibration protocol (in module htmd.protocols.equilibration v2) was subjected to the following modifications: the integration step was set to 2 fs, and constraints of 0.1 kcal/mol on the heavy atoms of ATP were added to the default constraints. The default production protocol (in module htmd.protocols.production v6) was used as is.

### Methods for analysis

#### Angle between two domains

**Figure 1:**
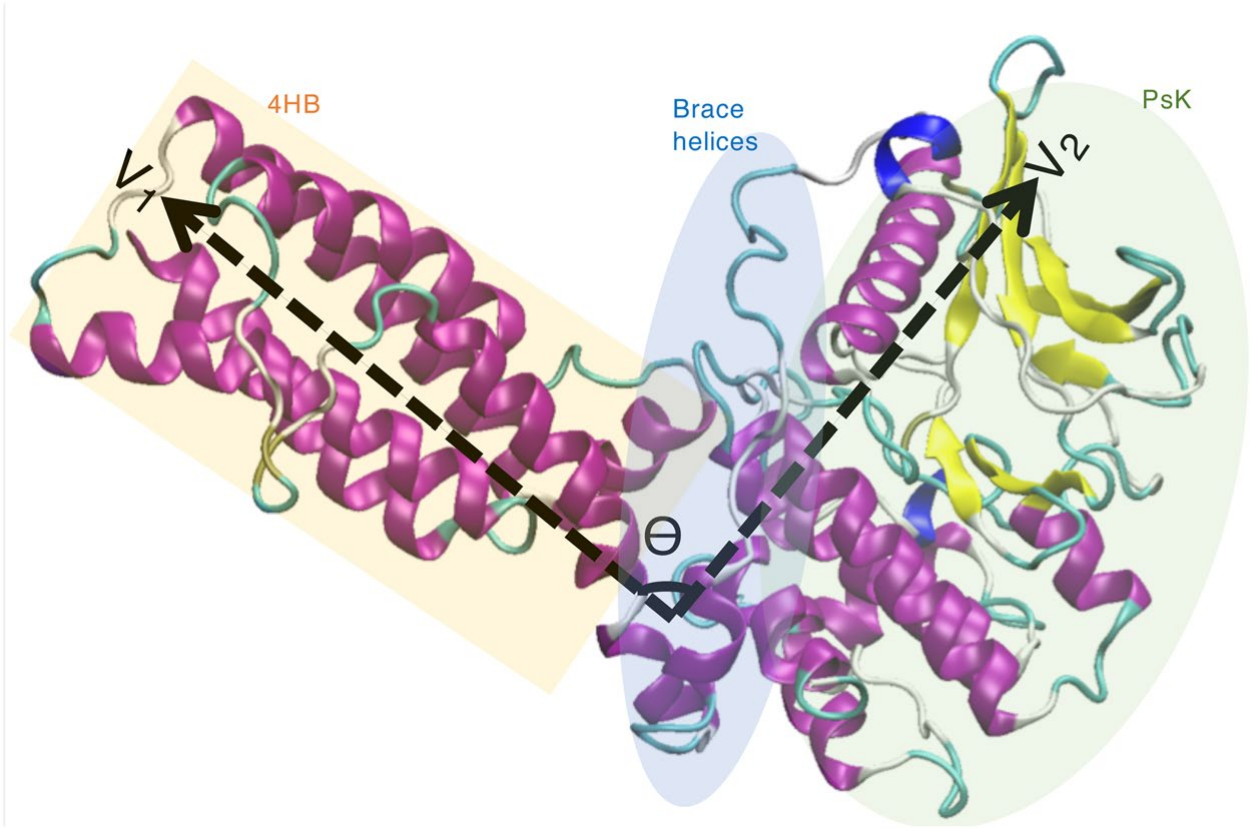
Pictorial representation of vectors V_1_ and V_2_ representing the N-terminal helical bundle and the pseudokinase domains, respectively. N-terminal helical bundle (4HB) is highlighted in orange color, Brace helices in blue and pseudokinase domain (PsK) in green color.

Angle θ between the vectors, V_1_ and V_2_ was calculated by CPPTRAJ utility of Ambertools^41^ (Fig. 1). The vectors were defined by taking the center of mass of N, CA and C atoms between residues (1–4 and 140–145) for vector V_1_ and between residues 145–149 and 468–472 for vector V_2_.

#### Alpha helical content

The alpha helical percentage composition of each residue averaged over the entire simulation length for the three systems was calculated using CPPTRAJ. The percentages were calculated with respect to the starting structure of each simulation as the reference.

#### Cross correlation matrices

The cross-correlation matrices were calculated for backbone Cα atoms averaged over the entire trajectory using CPPTRAJ utility.

#### Radial distribution function (g(r))

The g(r) plots were obtained using VMD. The pairwise interaction energies between residues Lys157 and Cys86 were calculated using CPPTRAJ.

Salt bridge interactions are obtained through VMD and all the hydrogen bond interactions are obtained using CPPTRAJ utility of Amber.

## Results

We generated a total of 3 μs simulation data for apo, phosphorylated and NSA bound hMLKL systems using classical MD simulations. The data were analyzed by CPPTRAJ utility of AmberTools18^41^ and VMD 1.9.3^42^ software. This section discusses results of the analysis of MD trajectories.

### Angle between two domains

Our simulations were able to capture wide inter-domain motions between N-terminal 4HB and PsK domains. The 4HB domain existed in cis state with the PsK domain in the starting conformation in all the three systems but the angle between the two domains fluctuated to a great extent, especially in apo and phosphorylated forms. The angular change between the N-terminal 4HB and PsK domains was measured in terms of angle θ as shown in Fig. 1.

**Figure 2:**
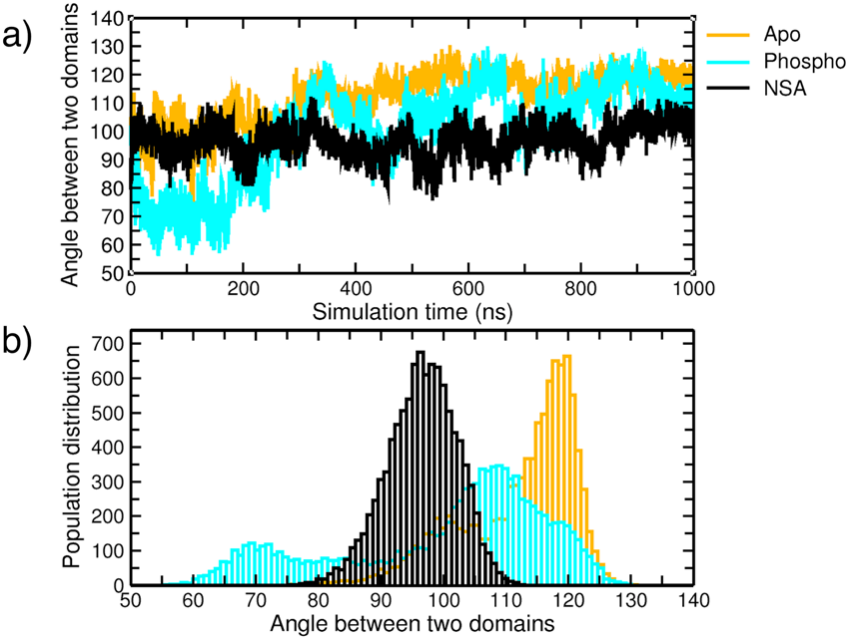
(**a**) represents calculated angle between the 4HB and the PsK domain (as shown in Fig. 1) for *apo*, phospho and NSA bound MLKL simulations. (**b**) Shows the histogram of the top panel plot showing population distributions based on angle.

Figure 2a shows the angle θ between 4HB and PsK domains for the entire simulation length for apo, phosphorylated and NSA bound MLKL simulations. NSA bound MLKL simulations show only slight perturbation of the angle during the entire trajectory (96.59 ± 5.5), whereas the angular deviation is quite large for apo and phosphorylated MLKL, (112 ± 9.01 and 100 ± 16.5, respectively). To obtain a clear understanding of the angular perturbation, histograms (as shown in Fig. 2b) were generated from the time series plot of the angular motion between the two domains. As can be clearly seen from the histogram plot, NSA bound simulations have a very narrow angular distribution with a single peak around ~96° while apo and phosphorylated MLKL simulations have two peaks with wider distributions. Although the histograms for both apo and phosphorylated simulations are showing two peaks, the two peaks occur at different angles with limited overlap of their angular distributions indicating sampling of different conformational spaces by these two MLKL states. Phosphorylated MLKL has much broader angular distribution while NSA binding causes the angular distribution to lock around one of the two peaks of apo MLKL. These results assert that NSA binding is locking the angle between the N-terminal 4HB and the PsK domains thereby restricting the unleashing of the helical bundle from the pseudokinase domain. On the other hand, apo and phosphorylated MLKL systems sample wider angular distributions suggesting a higher probability for 4HB domain to move away from PsK domain. This is the first simulation study to capture the locking of 4HB and PsK domains in NSA bound state.

### Alpha helical content

**Figure 3:**
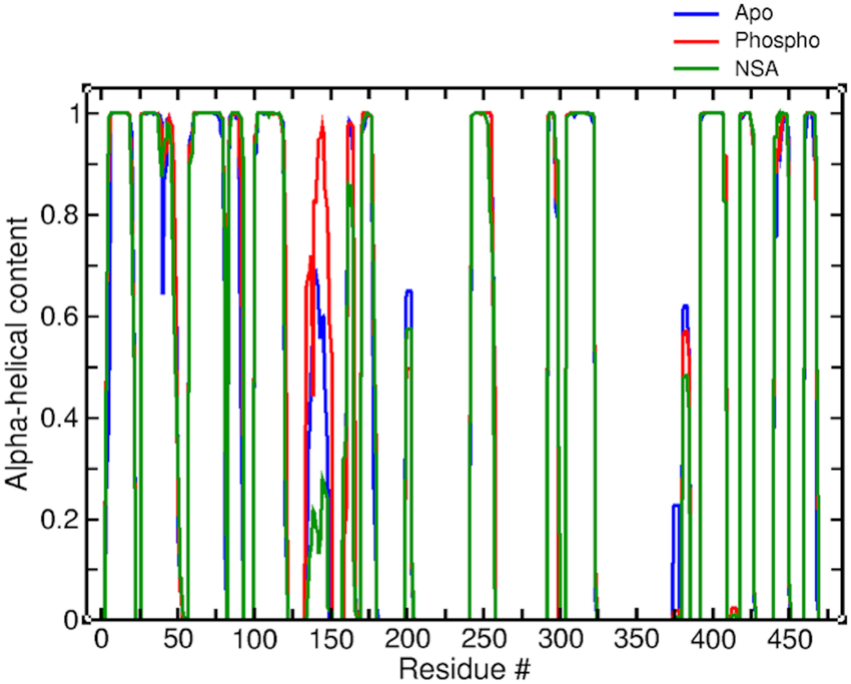
Alpha helical content in apo, phosphorylated (phospho) and NSA bound MLKL simulations averaged over the entire production run.

Next, we explored the impact of phosphorylation and NSA binding on secondary structure changes in MLKL structure. Similar to cross-correlation matrices, the difference in alpha helical percentages are mainly observed for the brace helices region (shown in Fig. 3). Average alpha helical percentage of the residues in the first brace helix is close to 35% in apo MLKL simulations while it shoots up to almost 65% in phosphorylated MLKL simulations and goes down to 10% upon NSA binding. The average alpha helical percentage of the entire brace helix region (first and second helices) show similar pattern: 34% in apo, 50% in phosphorylated and 19% in NSA bound MLKL simulations, respectively. The overall increase in the alpha helical content in the brace helix region upon phosphorylation is indicative of its increased stability as compared to apo form, which is quite likely representing the form of hMLKL that is suitable for oligomerization leading to necroptosis. Upon NSA binding, however, the stability of brace helices is lost. This is due to formation of cation-π interactions between brace helix residue Lys157

and the  aromatic ring in NSA which lead to changes in several interactions and orientation of brace helices with respect to 4HB and PsK domains (discussed further in later sections). Another segment that undergoes significant change in alpha helical content is comprised of residues 372–378 at the end of the activation loop in the PsK domain. The alpha helical percentage is close to ~20% in apo MLKL simulations, but it drops to ~1% in phosphorylated and NSA-bound MLKL simulations. We believe this change is reflective of the structural changes introduced by phosphorylation of Thr357 (TPO357) and Ser358 (SEP358) in the PsK domain. It is clear that the most dominant effects of phosphorylation and NSA binding are on the secondary structure composition of the brace helices suggesting that brace helices are mediating the interactions between 4HB and PsK domains.

### RMSD and RMSF

**Figure 4:**
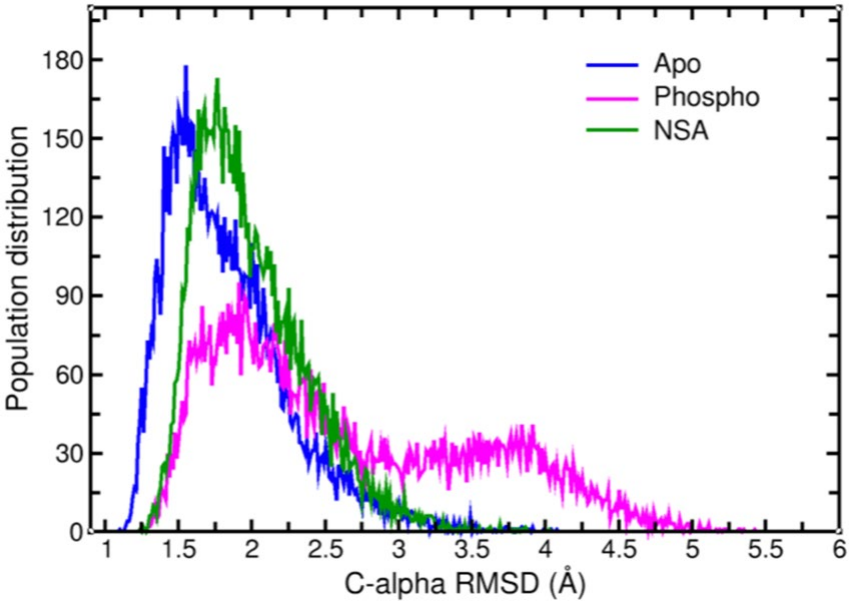
Distribution profile of  Cα RMSD (Å) for apo (blue), phosphorylated (magenta) and NSA bound (green) simulations.

Root mean square deviation (RMSD) (Å) is the simplest method to  assess the structural variability of the protein in different states. We plotted the  Cα RMSD of hMLKL protein for the entire simulation length for apo, phosphorylated and NSA bound states using the average structure from the simulation as the reference point. Figure 4 shows the histogram of the observed RMSD plots for apo, phosphorylated and NSA bound simulations. It can be observed from Fig. 4 that the RMSD distribution for apo (blue curve) shows a single uniform distribution with a peak around 1.5 Å. On phosphorylation, we observe two distinct distributions (magenta curve) with peaks at 1.95 Å and 3.8 Å, respectively. On NSA binding, only a single peak at 1.75 Å was observed. These observations are consistent with the angle distribution plots shown in Fig. 2. As shown earlier, the phosphorylated MLKL has two distinct populations. Interestingly, the average  Cα RMSD of NSA bound MLKL is slightly higher than that of Apo MLKL, because NSA causes much larger local mobility of residues around Cys-86 and Asp-144 (see later) in the first brace helix region. The time series plot is added in the Supporting Information in Fig. S12.

**Figure 5:**
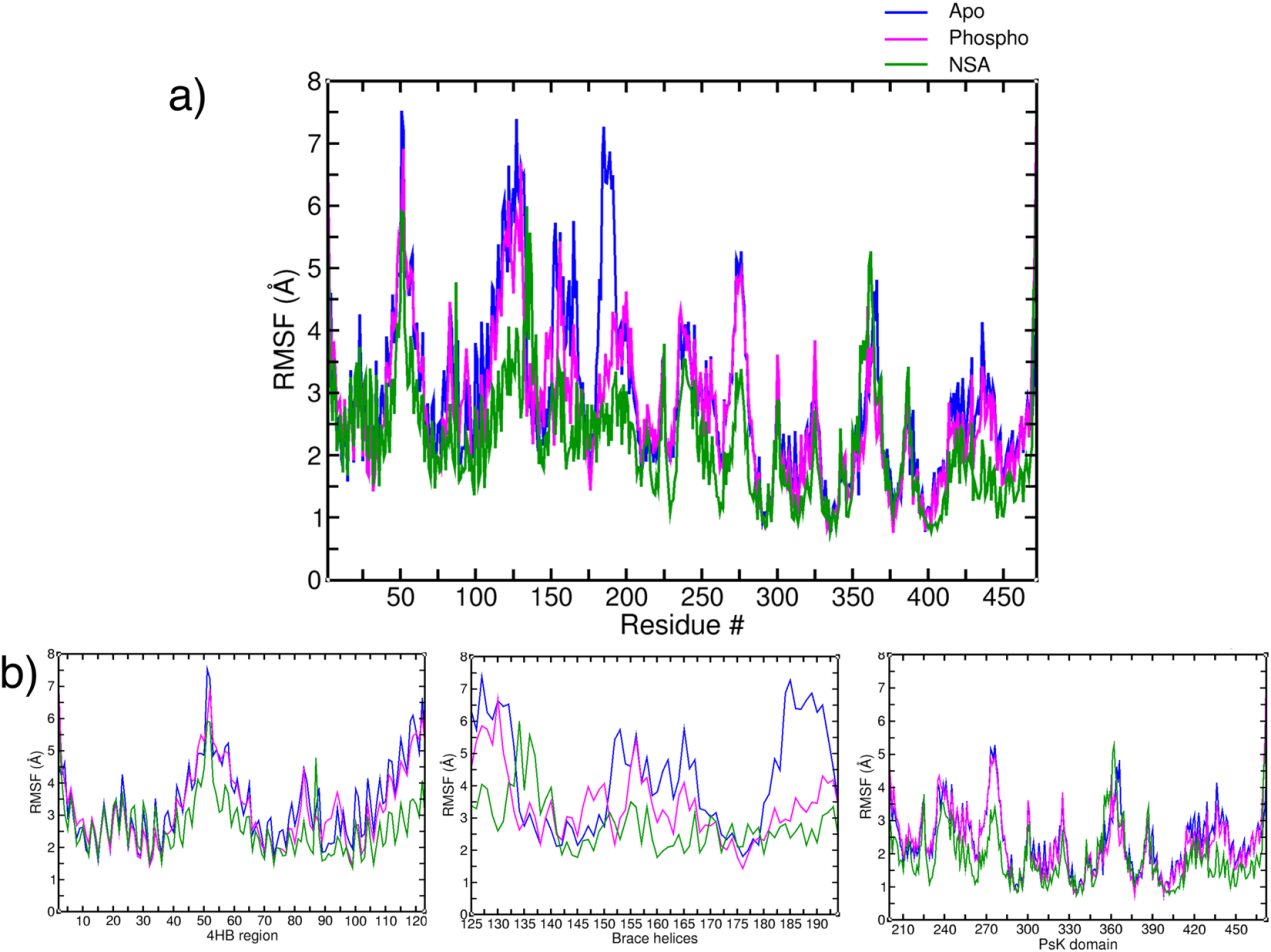
(**a**) Root mean square fluctuations (Å) of each residue in Apo (blue), Phosphorylated (magenta) and NSA bound (green) simulations. (**b**) Domain wise decomposition of residues based on 4HB region (left), brace helices (center), and PsK domain (right).

Root mean square fluctuation (RMSF) (Å) analysis, a measure of the extent of Cα mobility in the protein, is shown in Fig. 5a for each hMLKL residue in apo, phosphorylated and NSA bound MLKL simulations. Figure 5b shows the RMSF distribution along each domain for the three different trajectories. In the 4HB region, overall RMSF of each residue is comparatively lower in NSA bound simulations as compared to apo and phosphorylated simulations, except for Cys86 for which NSA bound trajectory shows higher variance as compared to the other two cases (shown in Fig. 5b). Similarly, residues in the brace helices region are showing lower deviation for NSA bound simulations except for the part of the first brace helix region. Residues in second brace helix (155–170) are showing higher RMSF for both apo and phosphorylated simulations. The loop connecting the brace helices and PsK domain (180–195) is showing much higher deviation for apo simulations as compared to phosphorylated and NSA bound simulations. In the PsK domain, activation loop (351–372) is showing high RMSF for NSA bound and apo simulations but is low for phosphorylated simulations indicating stability of activation loop upon phosphorylation. N-terminal and C-lobe of PsK domain are showing similar trends with high RMSF (Å) in apo and phosphorylated MLKL simulations but it should be noted that overall RMSF of the N-terminal of PsK domain is higher than the C-terminus. Overall, highest RMSF is observed in the brace helices region.

### Cross correlation matrices

**Figure 6:**
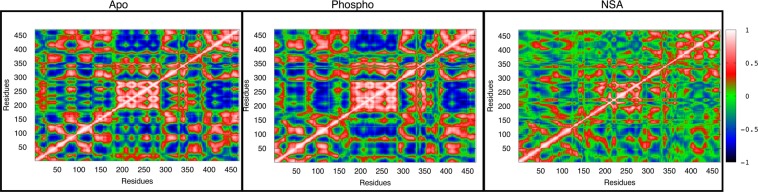
Cross-correlation matrices for Cα atoms in apo, phosphorylated and NSA bound MLKL simulations.

Cross-correlation matrices help us in investigating the effect of motion of a single residue on a network of residues. The calculated cross-correlation matrices for backbone Cα atoms from apo, phosphorylated and NSA bound MLKL simulations averaged over the entire trajectory are shown in Fig. 6. The matrices reveal several interesting differences in apo and phosphorylated states. Upon phosphorylation the positive correlation within the residues of the 4HB domain have become stronger as compared to the apo state. However, the positive correlation between the first and the second brace helices have weakened in phosphorylated MLKL. Also, the dynamics between the N-lobe of PsK and the first brace helix show stronger anti-correlation upon phosphorylation as compared to apo simulation indicating that they are moving in opposite directions. Interestingly, the A-loop shows neither a positive correlation nor an anti-correlation with the rest of the domains in phosphorylated MLKL as compared to apo, indicating that the phosphorylated A-loop is less mobile.

The cross-correlation matrix of NSA bound MLKL simulation is significantly different from both apo and phosphorylated MLKL as shown in Fig. 6. The strong correlation within the N-lobe of PsK domain has diminished upon NSA binding as compared to both Apo and phosphorylated MLKL. Overall, a lot of positive and anti-correlations are lost in the NSA bound simulation compared to both apo and phosphorylated MLKL simulations suggesting less conformational sampling and loss of mobility upon NSA binding which is consistent with the previous observation of interlocking of 4HB and PsK domains. We also performed principal component analysis (PCA) to look at the variance in the data. The PCA analysis is presented in Section S1 in Supplementary Information.

### Changes in brace helices

**Figure 7:**
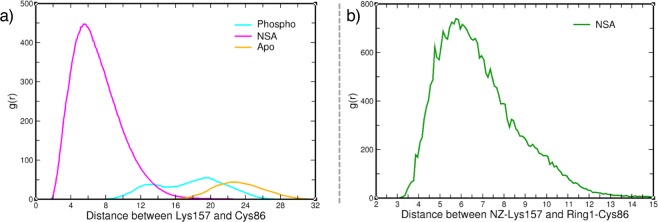
Radial distribution function (g(r)) between Lys157 and Cys86. The left panel (**a**) displays the g(r) between Lys157 and Cy86 for Apo, phosphorylated and NSA bound MLKL. (**b**) Shows the g(r) between NZ-Lys157 and  aromatic  ring (Ring1) of Cys86 for NSA bound simulations.

On further analysis of the key interactions of the residues in the brace helices that are affected upon phosphorylation and NSA binding, we observe that Lys157 of the second brace helix in the NSA bound state moved closer to Cys86 compared to apo or phosphorylated states as shown in the radial distribution function (g(r)) plot in Fig. 7a. The peak of the interaction in the NSA bound simulations is stronger and shorter (around 6 Å) compared to the other two simulations indicating the strengthening of the contact between Lys157 and Cys86. On closer evaluation, we observe that the interaction is mainly occurring between the terminal NZ atom of Lys157 and the aromatic ring (which is designated as Ring1) of NSA conjugated to Cys86 indicating a strong cation-π interaction (Fig. 7b). The time-series interaction energy plot for electrostatics and van der Waals (vdW) interaction for apo, phosphorylated and NSA bound MLKL is shown in Fig. S3 of Supplementary Information. The average interaction energies are listed in Table S1 of Supplementary Information. In the case of NSA-bound simulations, the electrostatics and vdW average interaction energies are much lower compared to the apo and phosphorylated state. Importance of Lys157 in necroptosis was also reported by Petrie *et al*.^13^. They observed that Lys157 crosslinks with Lys157 from another monomer in the tetrameric form and its mutation led to deficits in liposome permeabilization^13^. Our simulation data show that NSA binding flips the Lys157 sidechain and locks it in a cation-π interaction with the aromatic ring of NSA and makes it unavailable for crosslinking and tetramer formation.

**Figure 8:**
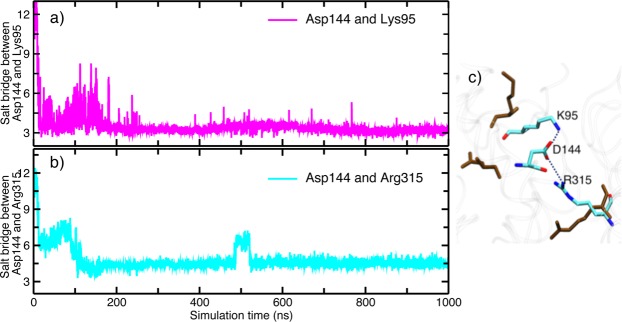
Salt bridge formation between brace helix residue Asp144 with N-terminal 4HB and PsK domain. (**a**) represents salt bridge formation between Asp144 and Lys95. (**b**) Represents salt bridge interaction between Asp144 and Arg315. Calculations were done with the VMD analysis tab^42^. (**c**) Shows the salt bridge interactions of Asp144 with Lys95 and Arg315 in NSA bound simulation (multi colored) superimposed with the residues from phosphorylated simulations (brown color).

Flipping of Lys157 causes major perturbations to the interactions in the rest of the brace helices region. For instance, Asp144 of the first brace helix near Lys157, is now freed to stabilize the interactions between N-terminal 4HB and PsK domains of MLKL: the carboxyl group of Asp144 forms a conserved salt bridge with the side-chain of Lys95 from the N-terminal 4HB (shown in Fig. 8a), and additionally with the guanidine group of Arg315 in the PsK domain (Fig. 8b). Figure 8c shows the interaction of residue Asp144 with Lys95 and Arg315 in phosphorylated and NSA bound simulations. These salt bridge interactions seem to persist throughout the NSA bound trajectory, while absent in both *apo* and phosphorylated MLKL states. Fig. S4 shows distances between Asp144-Lys95 and Asp144-Arg315 in apo and phosphorylated MLKL simulations. These residues are >10 Å apart in both apo and phosphorylated MLKL simulations, which suggests no salt bridge formation.

**Figure 9:**
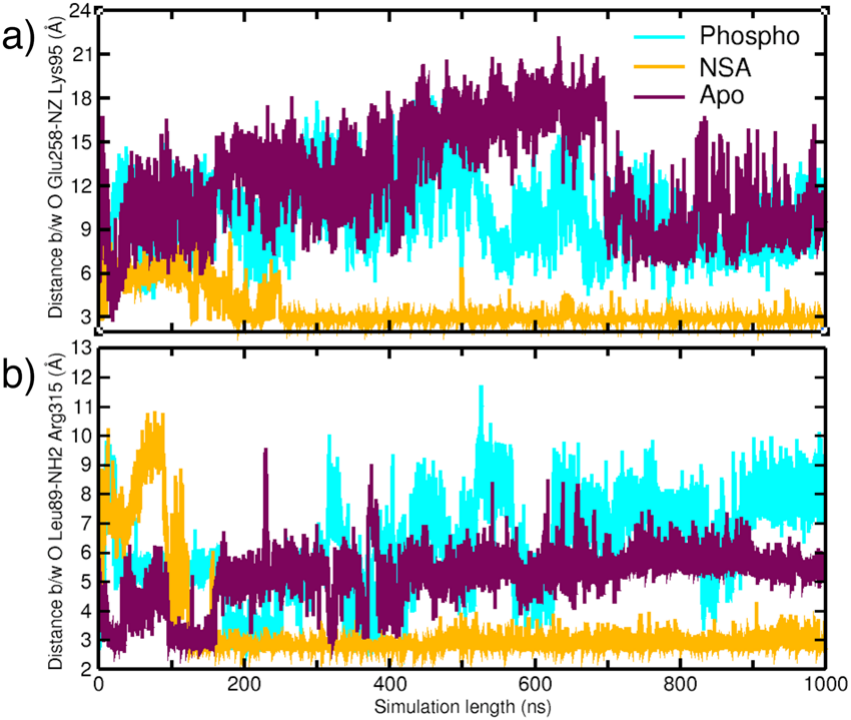
(**a**) H-bond interaction between carbonyl oxygen of Glu258 and NZ of Lys95 in *apo*, phosphorylated and NSA bound MLKL simulations. (**b**) H-bond interaction between carbonyl oxygen of Leu89 and NH2 of Arg315 in *apo*, phosphorylated and NSA bound MLKL simulations.

Another important salt bridge interaction is observed between a second brace helix residue Glu187 and Lys255 of α-C helix in PsK domain in the NSA bound simulation. This salt bridge was observed only in the beginning of the *apo* MLKL simulation (Fig. S5) but not in the phosphorylated MLKL simulation. Another weak salt bridge between a brace helix residue Glu171 and Lys305 from the PsK domain was formed upon NSA binding. Histograms of salt bridge formed between Glu171 and Lys305 for *apo*, phosphorylated and NSA bound MLKL simulations are shown in Fig. S6. Petrie *et al*. also identified Lys305 to be cross-linking with the same residue from another monomer and helping in MLKL oligomerization^13^. Salt bridges have been known to contribute to the protein stability^43^. Our analysis thus far suggests that upon NSA binding, several residues in brace helices are engaging in salt bridge formation with residues from PsK and 4HB domains. These newly established inter-domain interactions mediated by brace helices are opening avenues for new interactions between the 4HB and PsK domains. We observe new hydrogen bond formation between O-Glu258 from PsK domain and terminal NZ-Lys95 from 4HB domain in NSA bound MLKL simulations as shown in the Fig. 9a. This H-bond is formed at around 200 ns and persisted throughout the simulation only in NSA bound MLKL, but not in the *apo* or phosphorylated MLKL. Another H-bond was observed between carbonyl oxygen of Leu89 from 4HB and NH2 of Arg315 from PsK in NSA bound simulations as shown in Fig. 9b. It appears that these H-bond interactions between 4HB and PsK domains upon NSA conjugation are governed by the interactions of brace helices with 4HB and/or PsK domains.

**Figure 10:**
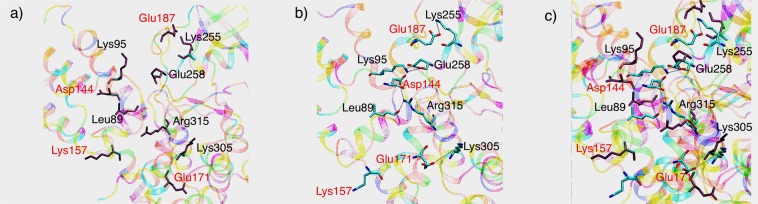
Positions and interactions of Leu89, Lys95, Asp144, Lys157, Glu171, Glu187, Lys255, Glu258, Lys305 and R315 in (**a**) phosphorylated simulations, (**b**) NSA bound MLKL simulations. (**c**) Overlay of these residues shown as sticks in both phosphorylated (shown in gray) and NSA bound simulations (colored). Braces helices residues are marked in Red color.

From Figs 8 and 9, we gather that upon NSA binding, MLKL protein is stabilized by a network of salt bridge and H-bond interactions between the residues of 4HB and PsK domains. Some of these interactions and the key residues are highlighted in Fig. 10. The interactions are shown for the last snapshot of the simulation for both phosphorylated and NSA bound systems. Positions of residues Glu171 and Lys305 in the phosphorylated and the NSA bound simulations are shown in Fig. 10. The residues interacting directly between 4HB and PsK domains are Leu89, Lys95, Glu258 and Arg315 as shown in Fig. 10. Lys95 and Arg315 are also interacting via a salt bridge interaction mediated by a brace helix residue Asp144 as shown in Figs 8 and 10. On closer observation of Fig. 10b,c, we find that Asp144 is positioned to act as a hub to form a network of interactions with residues Leu89, Lys95, Glu258 and Arg315 directly or indirectly (Fig. S7).

We surmise that these residues collectively are stabilizing the interactions between 4HB and PsK domain upon NSA conjugation as shown in Fig. 10b,c. None of these interactions are observed in phosphorylated MLKL simulations as residues are positioned differently (shown in Fig. 10a). From our analysis, we have observed several residues which are participating in inter-domain interactions and keeping the MLKL conformation locked upon NSA binding. We have also observed new interactions established by brace helices’ residues Asp144, Lys157, Glu171 and Glu187 upon NSA binding. These residues are annotated in red in Fig. 10. The new interactions formed by the above-mentioned residues of brace helices are changing conformation and mediating interactions between 4HB and PsK domains thereby stabilizing and locking the MLKL conformation upon NSA binding. Overall, we observe two main effects of NSA binding on hMLKL: (1) locking of the relative orientation of 4HB and PsK domains by the formation of several new inter-domain interactions and (2) prevention of Lys157 (key 4HB residue) to participate in cross-linking for oligomer formation.

### Changes within PsK domain

**Figure 11:**
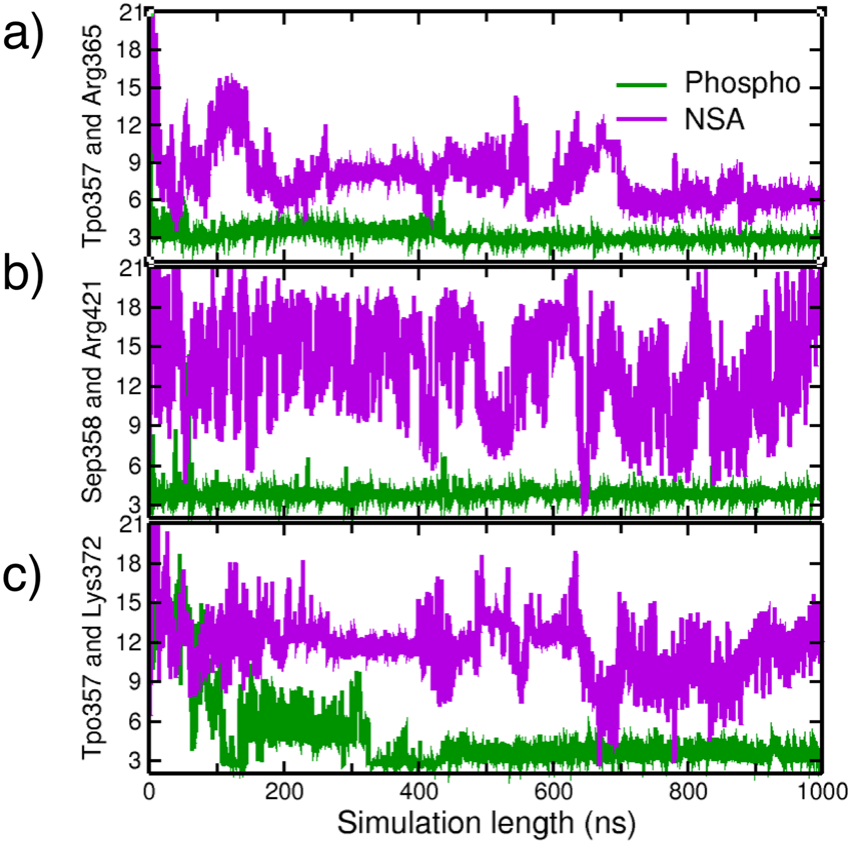
Salt bridge between (**a**): Tpo357 and Arg365, (**b**): Sep358 and Arg421, (**c**): Tpo357 and Lys372 in phosphorylated and NSA bound MLKL simulations.

Phosphorylation state of a kinase determines its activity and regulation by modulation of its structure and stability^44^. The rigidification of the A-loop upon phosphorylation has been observed in PKA by the use of H/D exchange experiments^45^ and Src kinase by molecular dynamics simulations^46^. Although MLKL is a pseudo kinase and lacks catalytic activity, its phosphorylation by RIP3 is important for its role in necroptosis. Our analysis thus far elucidated influence of phosphorylation on inter-domain mobility mediated via the motion of brace helices. In this section, we explore conformational changes observed within the PsK domain of MLKL upon phosphorylation and how or whether NSA binding is affecting those structural changes. Our analysis shows that upon phosphorylation of MLKL at Thr357 (TPO357) and Ser358 (SEP358), the interactions between the residues of the A-loop are stabilized and several other residues in the N and C lobes of the PsK domain form multiple H-bonds and salt bridges. Fig. S13 highlights the changes in activation loop interactions between phosphorylated and NSA bound MLKL simulations. This enhanced stability of the A-loop is not observed in *apo* form and is lost in the NSA bound MLKL simulations. Quite strong salt-bridge interactions between phosphoserine-Lysine in a helix-coil have been previously reported because of the negative charge imparted by phosphate group^39^. Figure 11 lists the salt bridge interactions formed by TPO357 and SEP358 with the other PsK domain residues in our phosphorylated MLKL simulations. Figure 11a,c show a persistent intra A-loop salt bridge interaction between TPO357- Arg365 and TPO357-Lys372. These salt bridge interactions are quite strong in the case of phosphorylated MLKL but lost when NSA conjugates to MLKL. On the other hand, in NSA bound simulations, we observe that A-loop is more flexible and Arg365 is interacting with Glu213 (P-loop residue) as shown in Fig. S8.

Additionally, we observe other intra-molecular interactions within the activation loop that are more stable in phosphorylated MLKL but do not exist or are lost in *apo* and NSA bound MLKL. In phosphorylated MLKL simulations, we identify a network of residues that is interacting and stabilizing the A-loop dynamics such as salt bridge formation between the gatekeeper residue GLu351 with Lys354 and Glu213  with  Lys354 (shown in Figs S9 and S10). All these interactions are rigidifying and stabilizing the A-loop upon phosphorylation. In NSA bound simulations, this stabilization of the A-loop is disrupted probably due to the conformational changes induced in brace helices and N-lobe of PsK domain, and the A-loop is less constrained.

We also observe that the interactions between the C-lobe of PsK domain and A-loop are weakened upon NSA binding, which were quite strong in phosphorylated MLKL. Figure 11b shows a salt bridge formation between Sep358 and Arg421 in phosphorylated MLKL simulations, which is lost upon NSA binding. Fig. S11 also shows a strong H-bond formation between carbonyl oxygen of SEP358 and side chain OG1 of Ser417 in the case of phosphorylated hMLKL simulations. All the critical salt-bridges and H-bonds discussed in the text are listed in Tables S2 and S3 in Supplementary Information. The structural changes observed in brace helices and the formation of new interactions between 4HB, brace and PsK domains upon NSA binding appear to be responsible for higher flexibility of A-loop negating its stabilization imparted by phosphorylation.

## Discussion

Mixed lineage kinase domain-like (MLKL) protein has emerged as a key contributor in necroptosis. MLKL gets activated by RIP3 which phosphorylates threonine and serine on the activation loop in the pseudokinase domain. Upon activation, the 4HB domain unleashes from the PsK domain, gets oligomerized and permeates through the plasma membrane causing cell death. Our goal in this study was to understand two main questions: (1) why phosphorylation of MLKL is important for necroptosis? and (2) how NSA binding on the 4HB domain is preventing necroptosis? Our simulations show increased inter-domain flexibility of phosphorylated MLKL as compared to *apo* or NSA bound MLKL while maintaining stable conformation of 4HB bundle that is suitable oligomerization and required for necroptosis. We also observe that activation loop of MLKL gets rigidified upon phosphorylation similar to other catalytically active kinases, although MLKL is a pseudo kinase and does not perform catalytic function. This structural ordering of activation loop is lost upon NSA binding negating the effect of phosphorylation. The NSA bound simulations showed the locking of the 4HB and PsK domains which was not seen in *apo* or phosphorylated simulations. We hypothesize that locking of the two domains is hindering the flexibility required for oligomerization of 4HB domain. These major changes are triggered by flipping of Lys157 to form cation-π interactions with the  aromatic ring of NSA covalently conjugated to Cys86, leading to loss of helicity in brace helices and its increased flexibility. Motion of brace helices facilitate formation of several new interactions between 4HB domain and the α-C helix region of the PsK domain upon NSA binding: the residues of brace helices (Asp144, Lys157, Glu171 and Glu187) interact with both 4HB and PsK domain residues directly or indirectly. For example, Asp144 is re-positioned as a hub to establish new direct or indirect interactions between Leu89, Lys95, Glu258 and Arg315 as shown in Fig. 9. All these interactions result in locking MLKL protein in a fixed conformation as opposed to flexible and open conformations in *apo* and phosphorylated forms.

This study provides new insights into the molecular mechanism of hMLKL protein’s non-catalytic function in necroptosis and rationalizes the previously unexplained phenomena related to successful inhibition of necroptosis by NSA, but not by compounds that bind tightly to the ATP pocket of MLKL. It is clear from our study that inhibitors that replace ATP in the ATP-binding pocket of the PsK domain of MLKL can neither lock the inter-domain conformation nor reorient key 4HB residues to prevent necroptosis as observed by NSA. This explains why the recently reported tight binders of MLKL ATP pocket do not show any anti-necroptotic activity^23^. This new understanding of the role and behavior of different domains of MLKL protein upon phosphorylation and NSA binding can help in designing better hMLKL inhibitors. Our study proposes that inhibitors that stabilize the locked conformation of the 4HB and PsK domains will prevent oligomerization and necroptosis.

## Supplementary information

Supplementary Information

Supplementary Dataset 1

Supplementary Dataset 2

## Data Availability

All data generated or analyzed during this study has been included in the published article or in the Supplementary Information. Any additional data is available upon request to the corresponding author.
