# Supplementary material for: Understanding allosteric interactions in hMLKL protein that modulate necroptosis and its inhibition
Source: Sci Rep. 2019 Nov 14;9:16853. doi: 10.1038/s41598-019-53078-5 (PMC6856060; doi:10.1038/s41598-019-53078-5)
Supplement: Supplementary file 1 — Supplementary Information [file 41598_2019_53078_MOESM1_ESM.pdf]

Supplementary Information

Understanding allosteric interactions in hMLKL protein that modulate necroptosis and its inhibition

Nupur Bansal, Simone Sciabola, and Govinda Bhisetti*

Biotherapeutic and Medicinal Sciences, Biogen, 225 Binney Street, Cambridge, MA 02142, United States of America

*Phone: +1 (617) 914-7273. E-mail: govinda.bhisetti@biogen.com

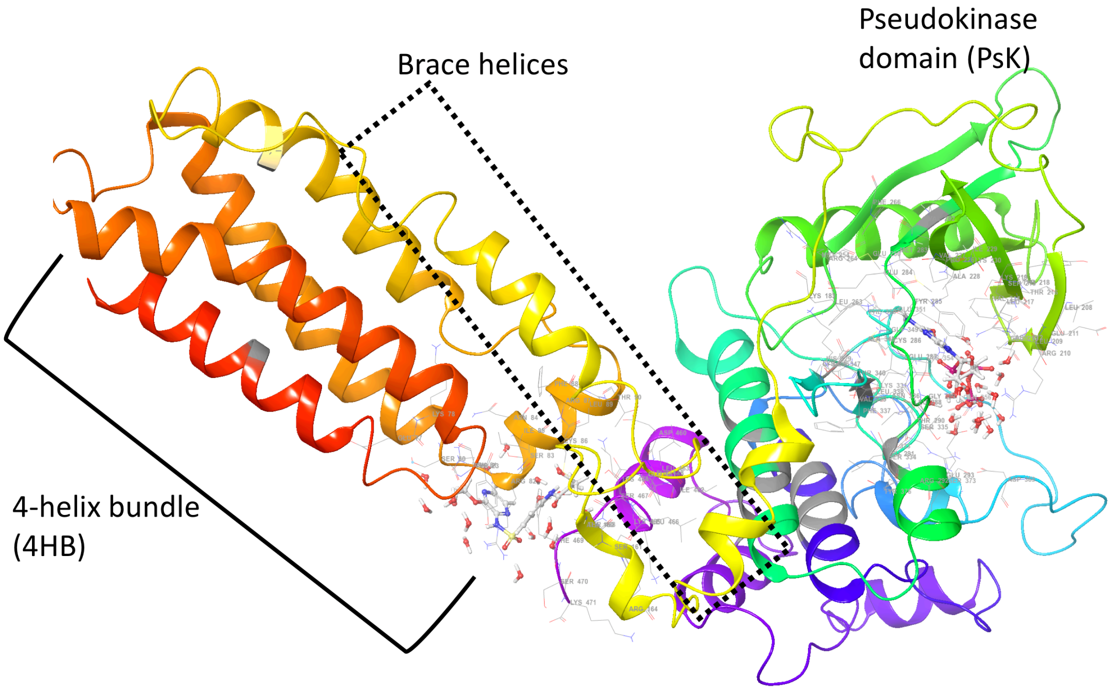

Figure S1. Structure of monomeric MLKL protein highlighting the N-terminal 4 helical bundle (4HB), Brace helices and C-terminal Pseudokinase (PsK) domains.

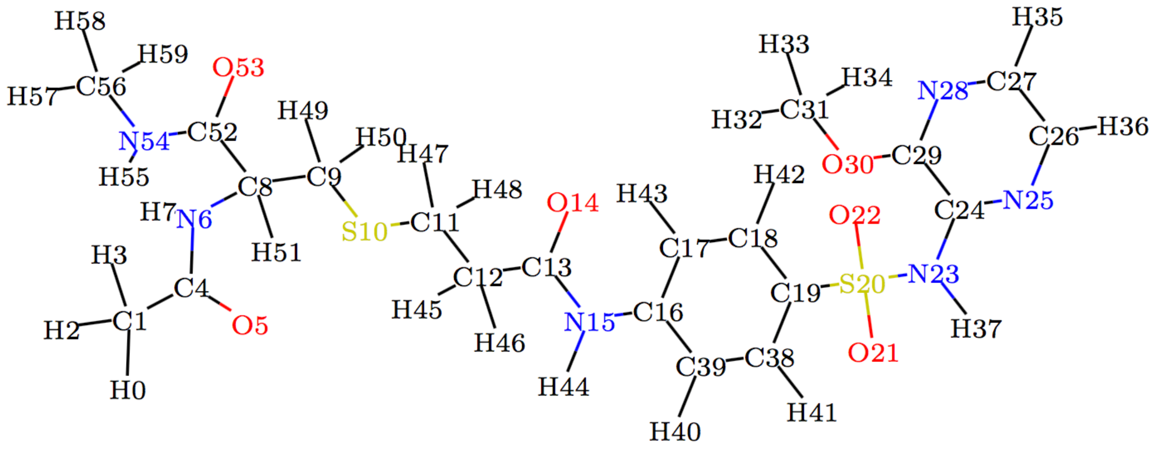

Figure S2. Molecular depiction of the CNS residue (cysteine conjugated with necrosulfonamide) capped with acetyl (ACE) and methylamine (NME). All bonds are depicted as single lines.

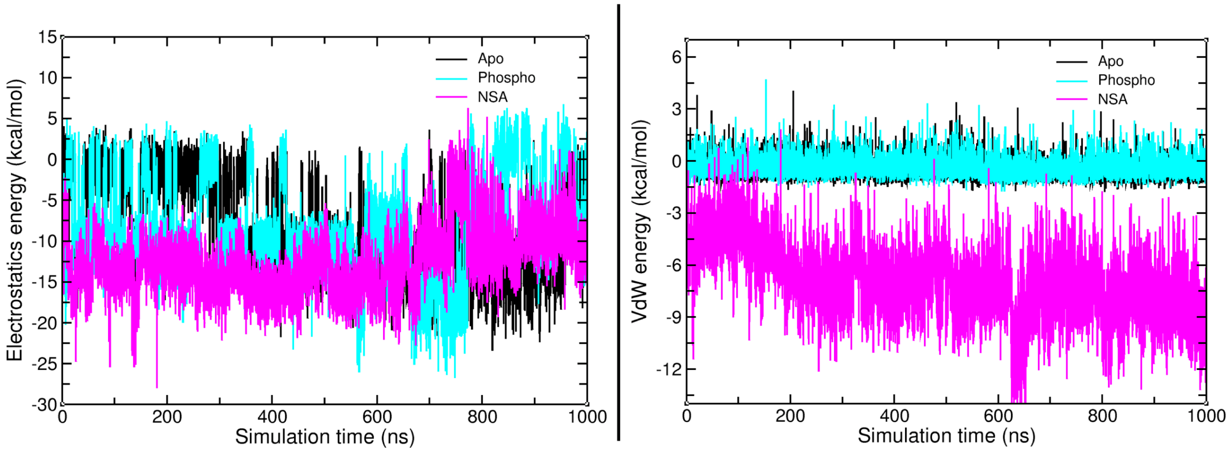

Figure S3. Pairwise interaction energy between Residues Cys86 and Lys157 in kcal/mol. Left: Electrostatics energy; Right: Vander Waal

Table S1. Average electrostatics and Vander Waal interaction energies (kcal/mol) between residues Lys157 and Cy86

	Apo	Phosphorylated	NSA-bound
Electrostatics (kcal/mol)	–8.45	–8.29	–12.64
VdW (kcal/mol)	–0.52	–0.39	–7.13

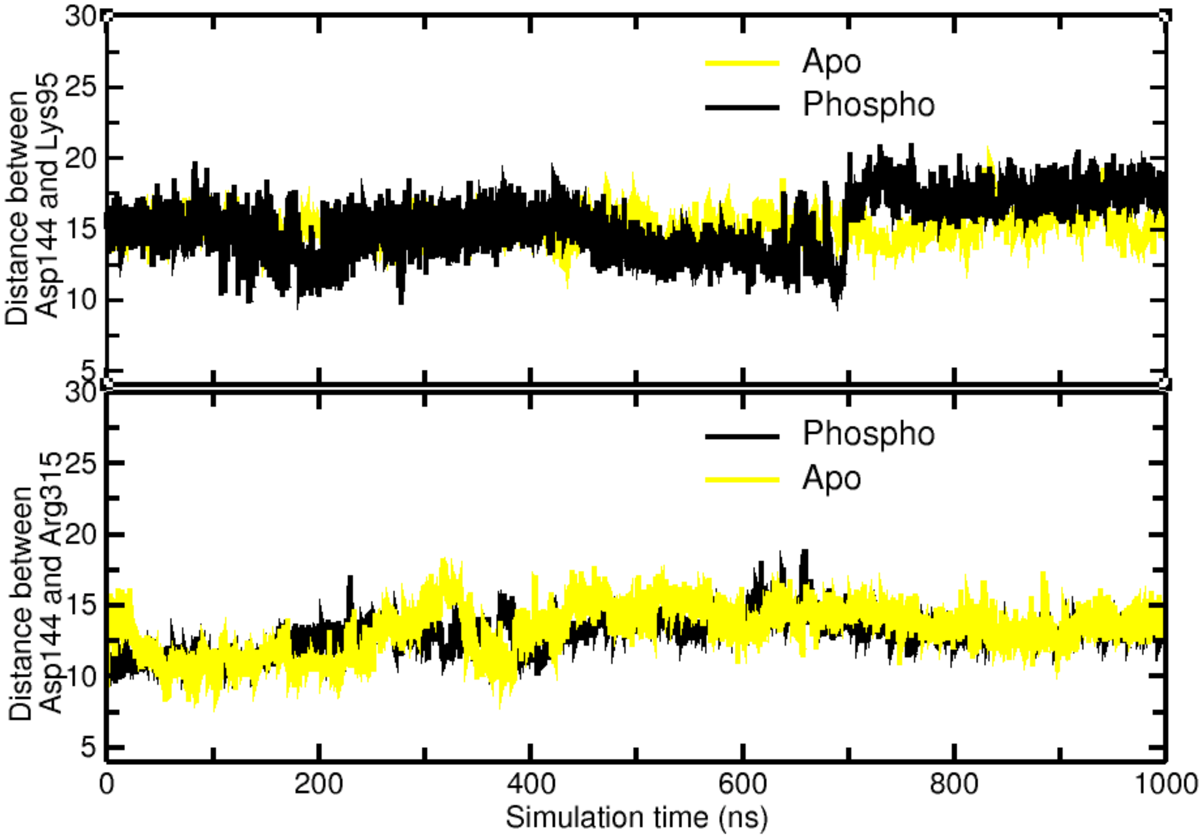

Figure S4. Distance between Asp144 and Lys95 and between Asp144 and Arg315 in Apo,and posphorylated MLKL simulations for the entire simulation. No salt bridge formation.

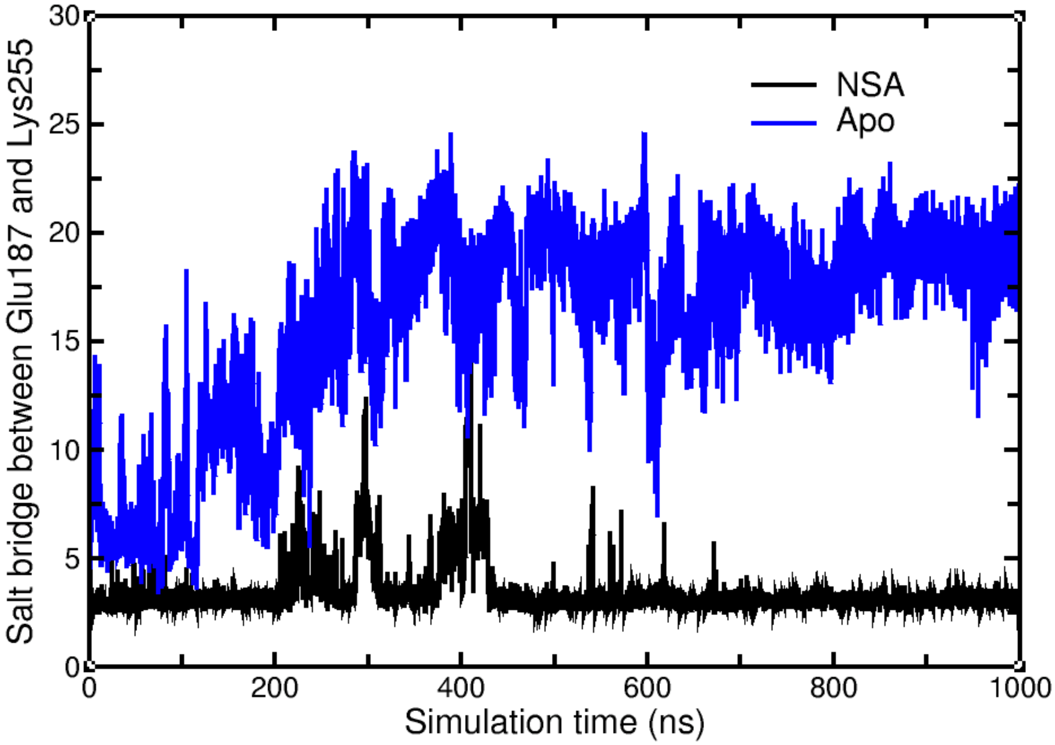

Figure S5. Salt bridge between Glu187 and Lys255 in Apo and NSA bound MLKL simulations for the entire simulation length.

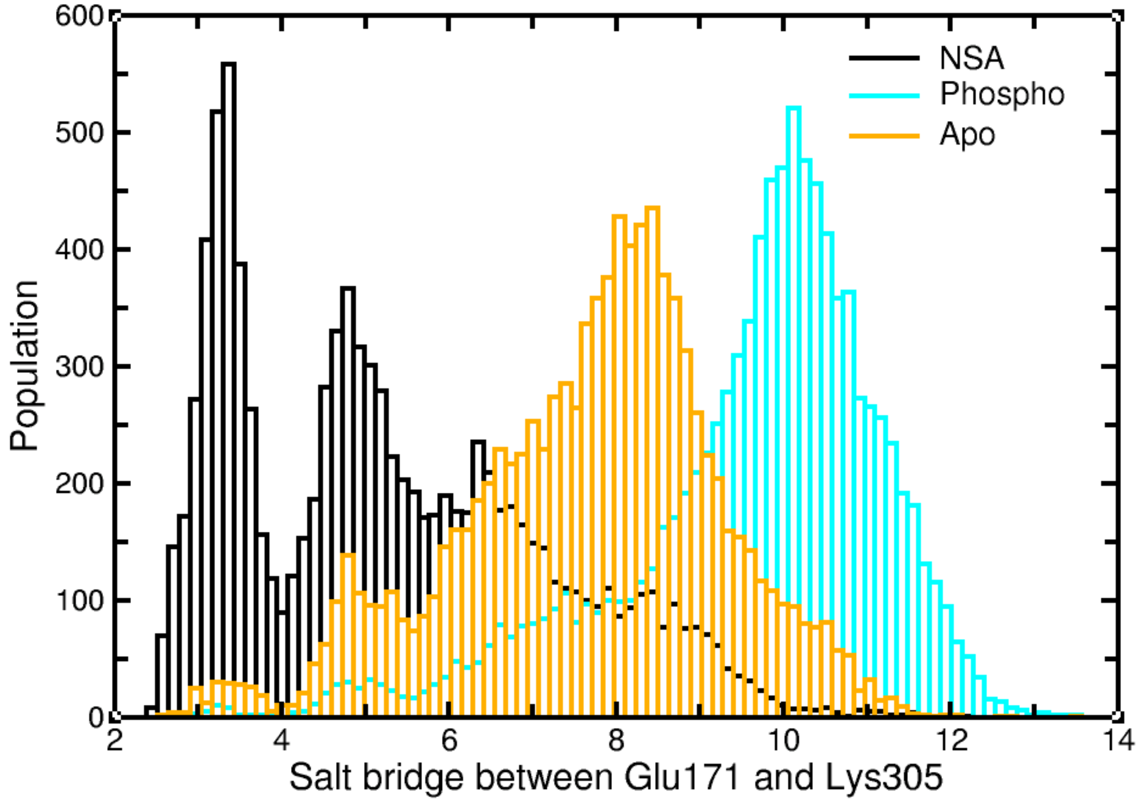

Figure S6. Histograms of salt bridge formations between Glu171 and Lys305 in apo, phosphorylated and NSA bound MLKL simulations. Histograms were evaluated for the entire simulation data.

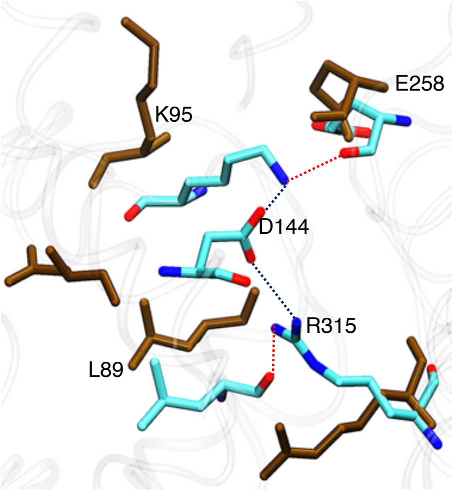

Figure S7. Positions and interactions of Asp144 with Leu89, Lys95, Glu258 and Arg315 in phosphorylated (brown) and NSA bound (colored) simulations. Asp144 has moved in the center in the NSA bound simulations and is directly/indirectly mediating interactions between 4HB and PsK domains. Black dotted lines indicate salt bridge interactions and red dotted lines represent hydrogen bond formation.

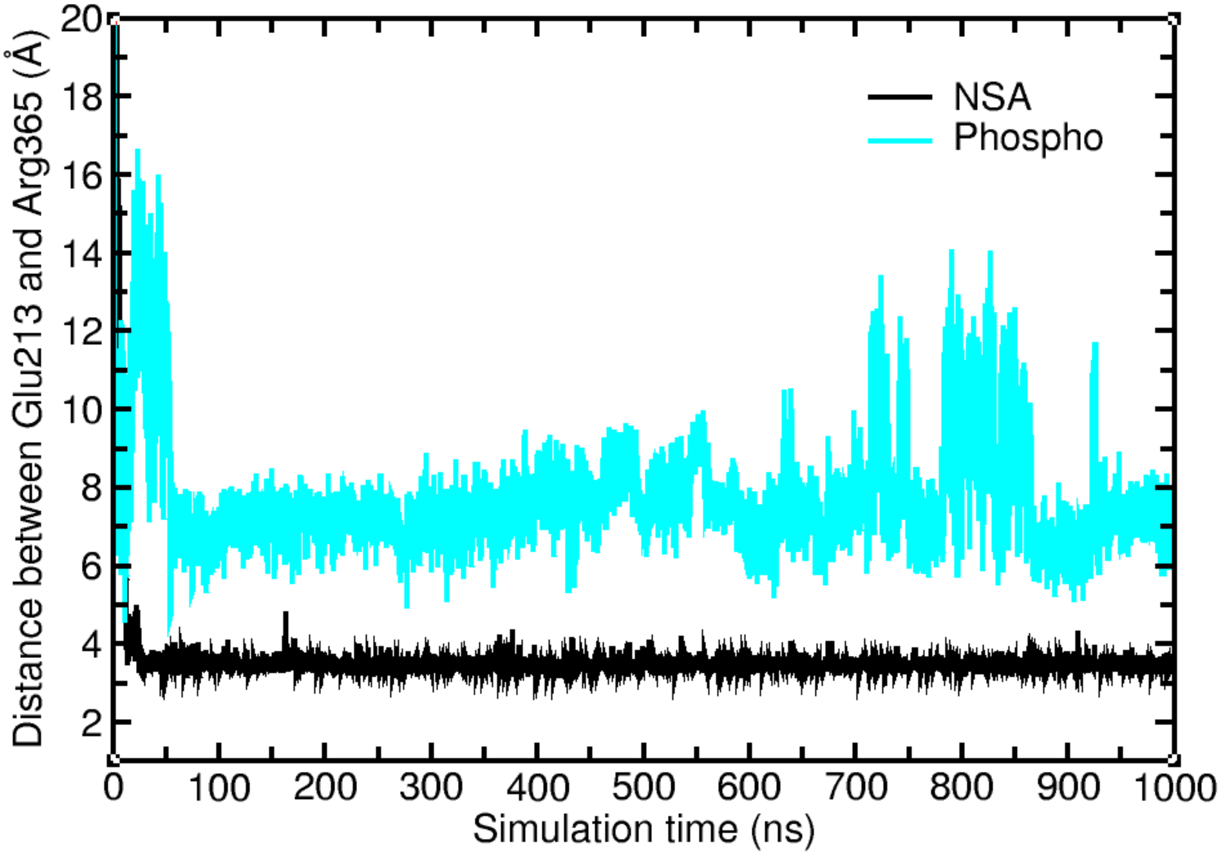

Figure S8. Salt bridge formation between Glu213and Arg365 in phosphorylated (phospho) and NSA bound MLKL simulations for the entire simulation length.

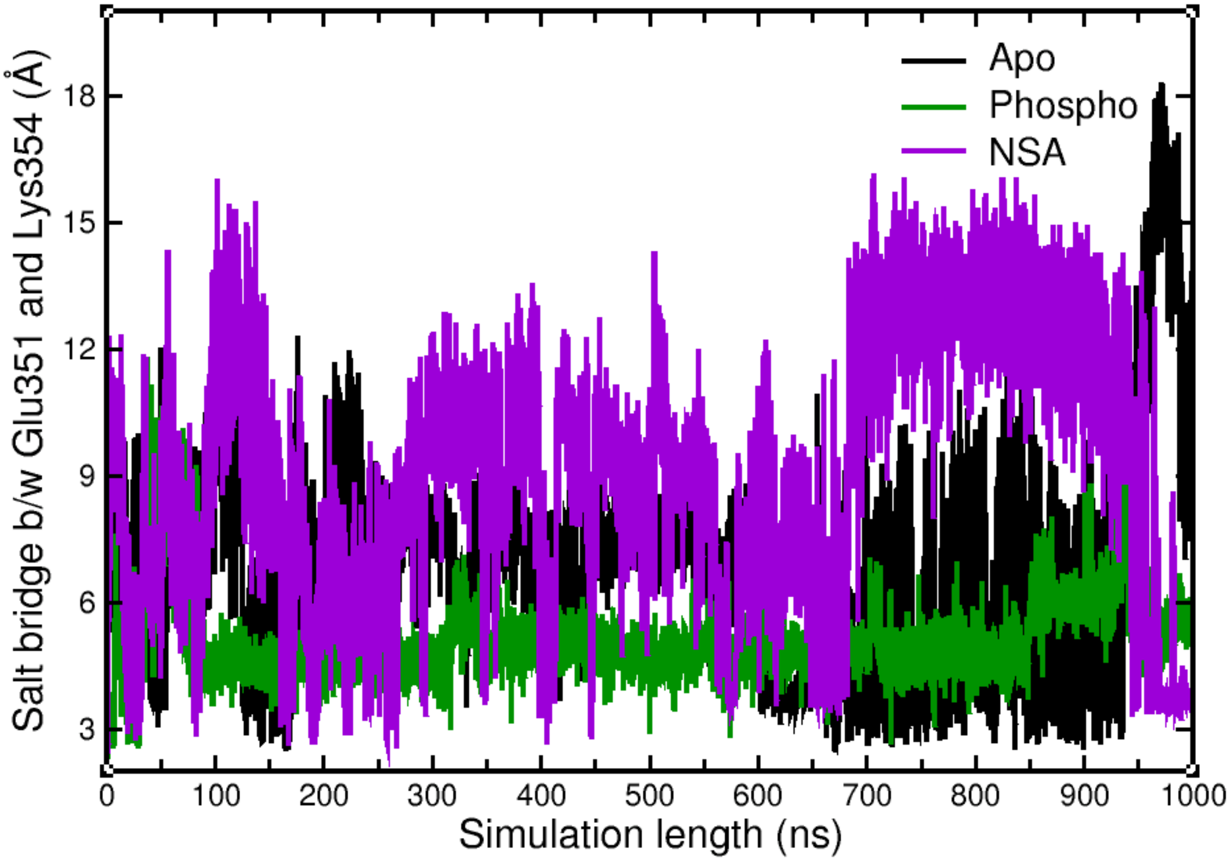

Figure S9. Salt bridge between GLU351 and LYS354 in apo, phosphorylated (phospho) and NSA bound MLKL simulations.

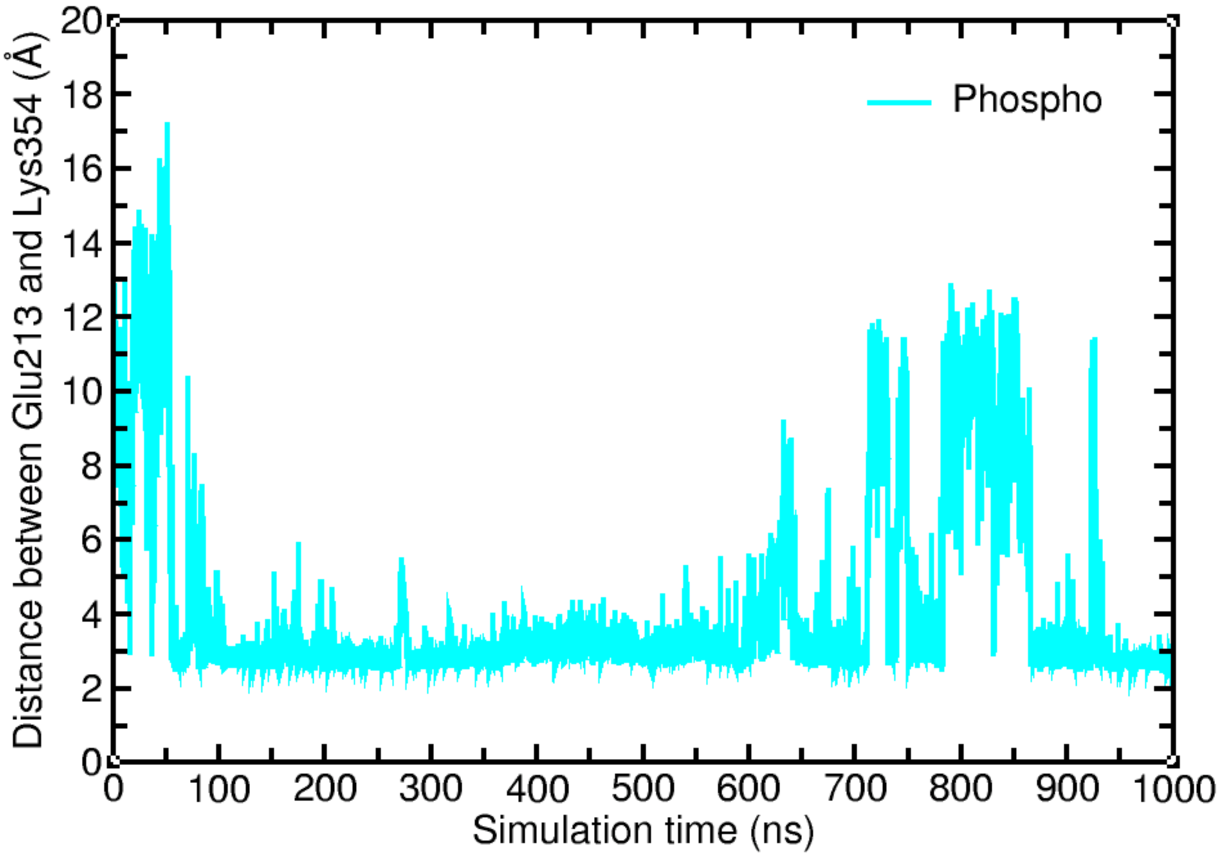

Figure S10. Salt bridge formation between Glu213and Lys354 in phosphorylated (phospho) MLKL simulations for the entire simulation length.

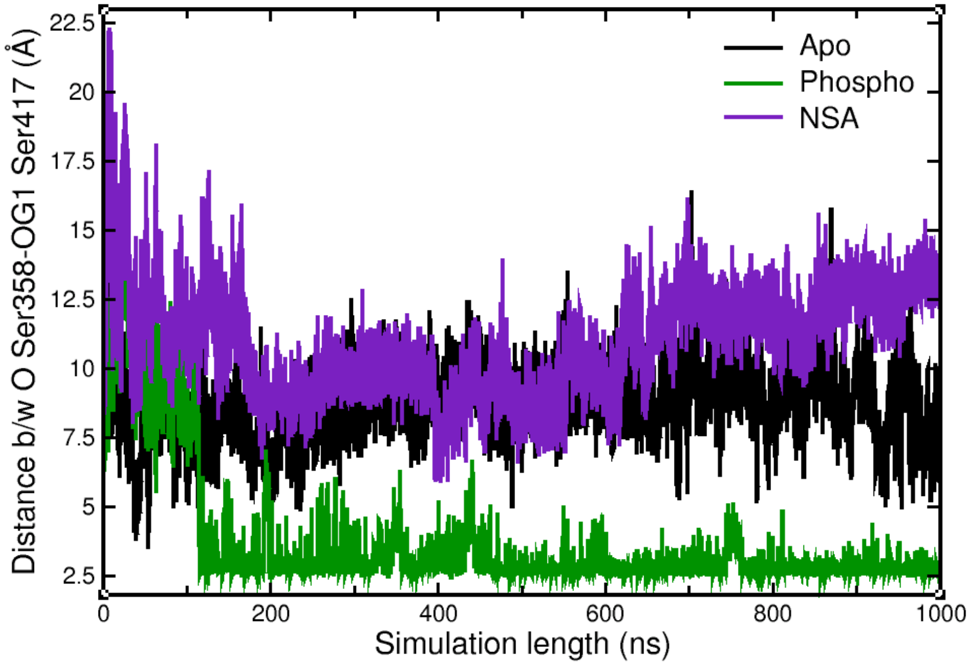

Figure S11. H-bond interaction between carbonyl O of Ser358 and OG1 of Ser417 in apo, phosphorylated (phospho) and NSA bound MLKL simulations.

Table S2. Lists all the critical salt bridges observed in Apo, Phosphorylated and NSA bound simulations. N/A stands for not applicable because Thr357 and Ser358 were not phosphorylated in Apo simulations and no salt bridge formation is possible.

Salt bridges

**Residue Pair**	**Position**	**Apo**	**Phosphorylated**	**NSA bound**
Asp144-Lys95	BraceHelix-4HB	Absent	Absent	Strong
Asp144- Arg315	BraceHelix-PsK	Absent	Absent	Strong
Glu187-Lys255	BraceHelix-PsK	Very Weak	Absent	Strong
Glu171-Lys305	BraceHelix-PsK	Very Weak	Very Weak	Medium
Tpo357-Arg365	PsK-PsK	N/A	Strong	Absent
Tpo357-Lys372	PsK-PsK	N/A	Strong	Absent
Glu213-Arg365	PsK-PsK	Absent	Absent	Strong
Glu351-Lys354	PsK-PsK	Weak	Strong	Very weak
Glu213-Lys354	PsK-PsK	Absent	Strong	Absent
Sep358-Arg421	PsK-PsK	N/A	Strong	Absent

Table S3. Lists all the critical H-bonds observed in Apo, Phosphorylated and NSA bound simulations.

H-bonds

Residue Pair	Position	Apo	Phosphorylated	NSA bound
O-Glu258;NZ-Lys95	PsK-4HB	Absent	Absent	Strong
O-Leu89;NH2-Arg315	4HB-PsK	Absent	Absent	Strong
O-SEP358;OG1-Ser417	PsK-PsK	Absent	Strong	Absent

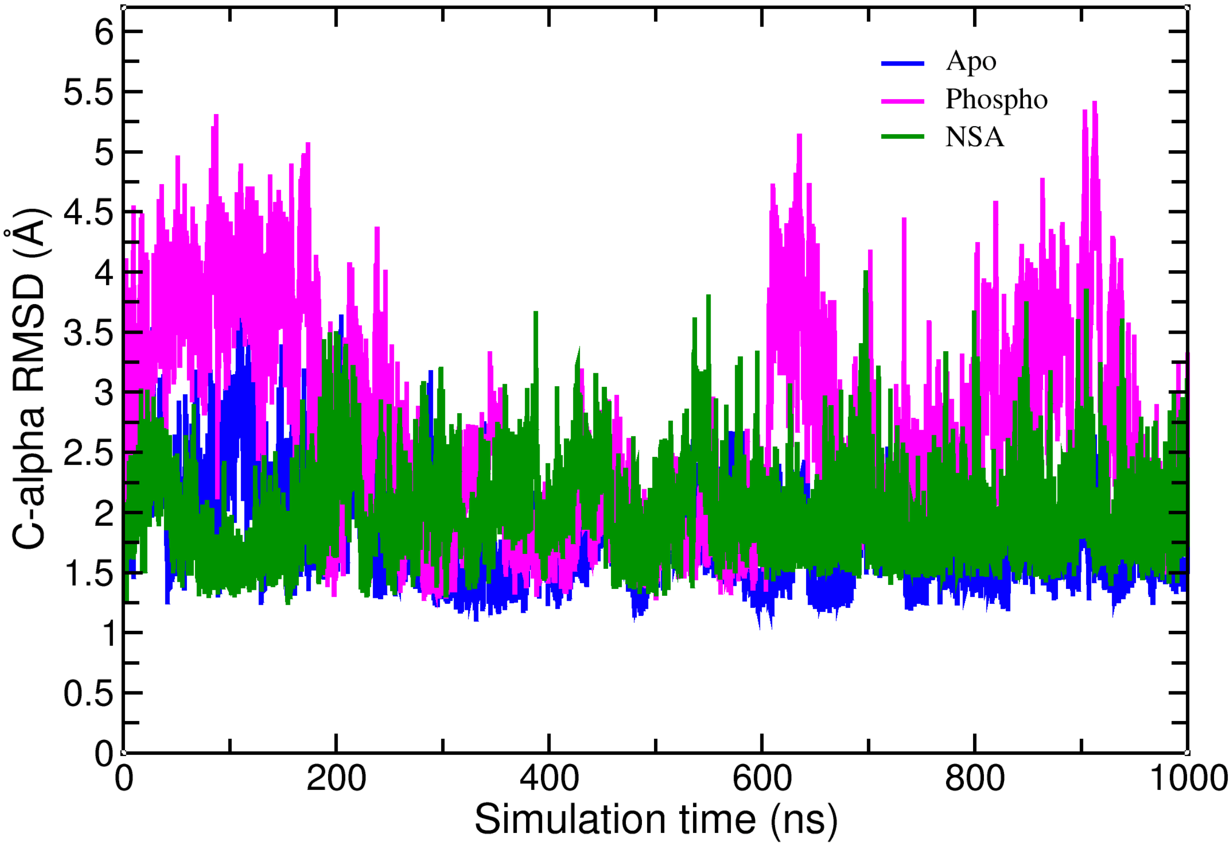

Figure S12. Time series plot of C-α RMSD (Å) for apo (blue), phosphorylated (magenta) and NSA bound (green) simulations for the entire 1 μs simulation length.

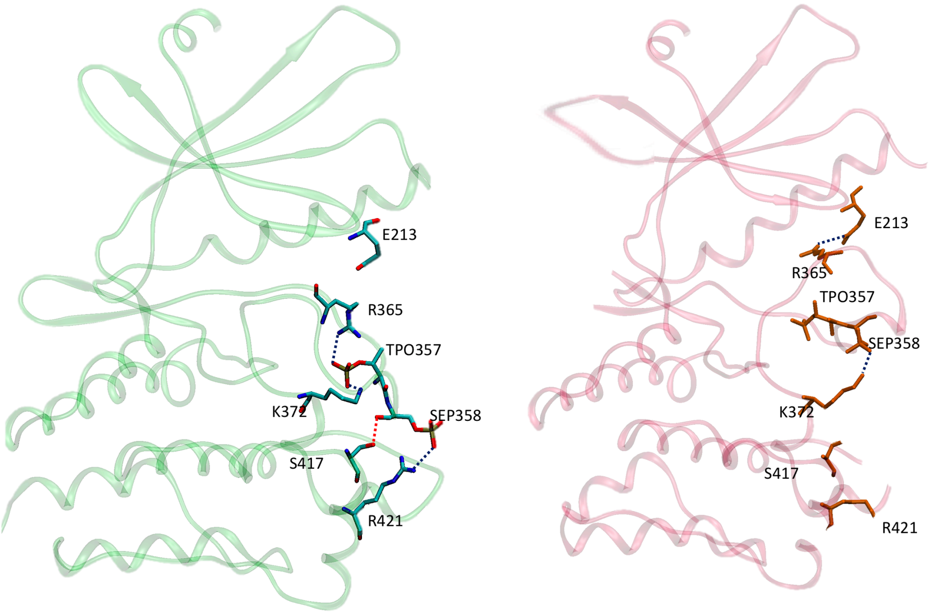

Figure S13. Psk domain of Phosphorylated MLKL (green) and NSA bound simulation (magenta) highlighting the interactions associated with activation loop in both the cases.

S.1. Principal Component Analysis (PCA)

We also performed the PCA of backbone Cα atoms in hMLKL protein for Apo, phosphorylated and NSA bound hMLKL simulations. PCA analysis gives us the extent of variance in conformational sampling and clusters the data based on structural similarity. The eigen vectors were obtained using CPPTRAJ and the trajectory was projected onto the largest principal components (PCs): PC1 and PC2. The two-dimensional density distributions of apo, phosphorylated and NSA bound simulations projected onto PC1 and PC2 are shown in Figure S14. It should be noted that the ranges of PC1 (x-axis) and PC2 (y-axis) are different in the three different simulations. Apo and Phosphorylated simulations data have somewhat similar ranges of PC1 and PC2, but not for NSA bound simulation. The density plot for NSA bound simulations clearly show two clear population distributions; one with high density and a second with a lower density. We overlaid the snapshots of MD trajectories from the two distributions and observed that

the 2 clusters differ in the activation loop and an N-terminal loop of PsK domain while the angle between the two domains remains in the closed conformation (see Figure S15 in supporting information). The apo simulations show multiple population distributions with two distinct major populations. The superposition of the representatives from the two major distributions from apo simulations showed structures with different domain angles in closed and open states of hMLKL (shown in Figure S16 in supporting information). PCA analysis of phosphorylated simulations is showing multiple population distributions (shown in center plot in Figure S14). The open state of hMLKL with the wider domain angle between 4HB and PsK is the population with maximum density.

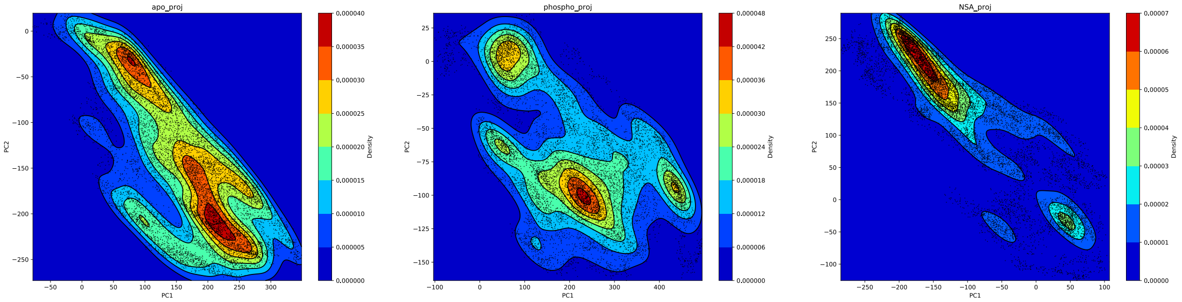

Figure S14. Two dimensional projection of the conformational space sampled by molecular dynamic simulations for Apo (left), Phosphorylated (center) and NSA bound (right) hMLKL simulations onto the two Principal components-PC1 and PC2.

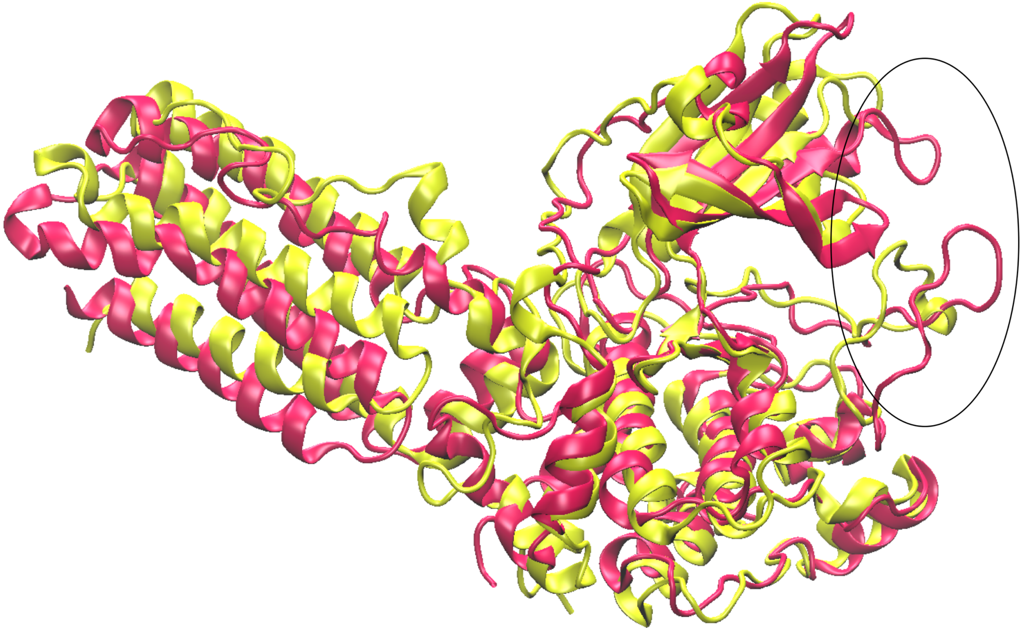

Figure S15. Superposition of the snapshots of two population distributions from NSA bound simulations based on the PCA analysis. The angle between the two domains is intact but the activation loop and P-loop in PsK domain has moved quite a bit.

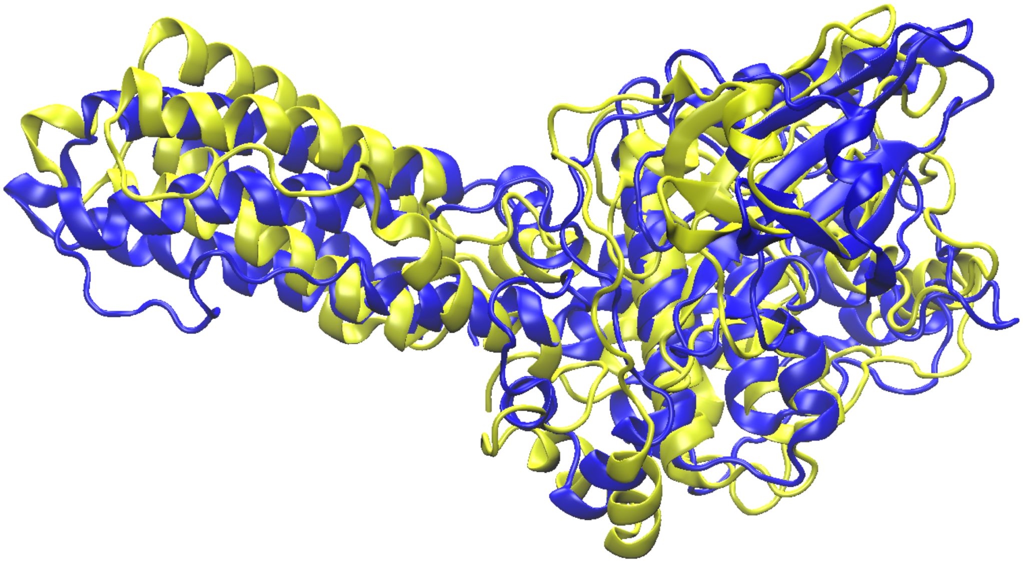

Figure S16. Superposition of the snapshots of two major population distributions from Apo bound simulations based on the PCA analysis.
